# Supplementary material for: Targeting brain lesions of non-small cell lung cancer by enhancing CCL2-mediated CAR-T cell migration
Source: Nat Commun. 2022 Apr 20;13:2154. doi: 10.1038/s41467-022-29647-0 (PMC9021299; doi:10.1038/s41467-022-29647-0)
Supplement: Supplementary file 1 — Supplementary Information [file 41467_2022_29647_MOESM1_ESM.pdf]

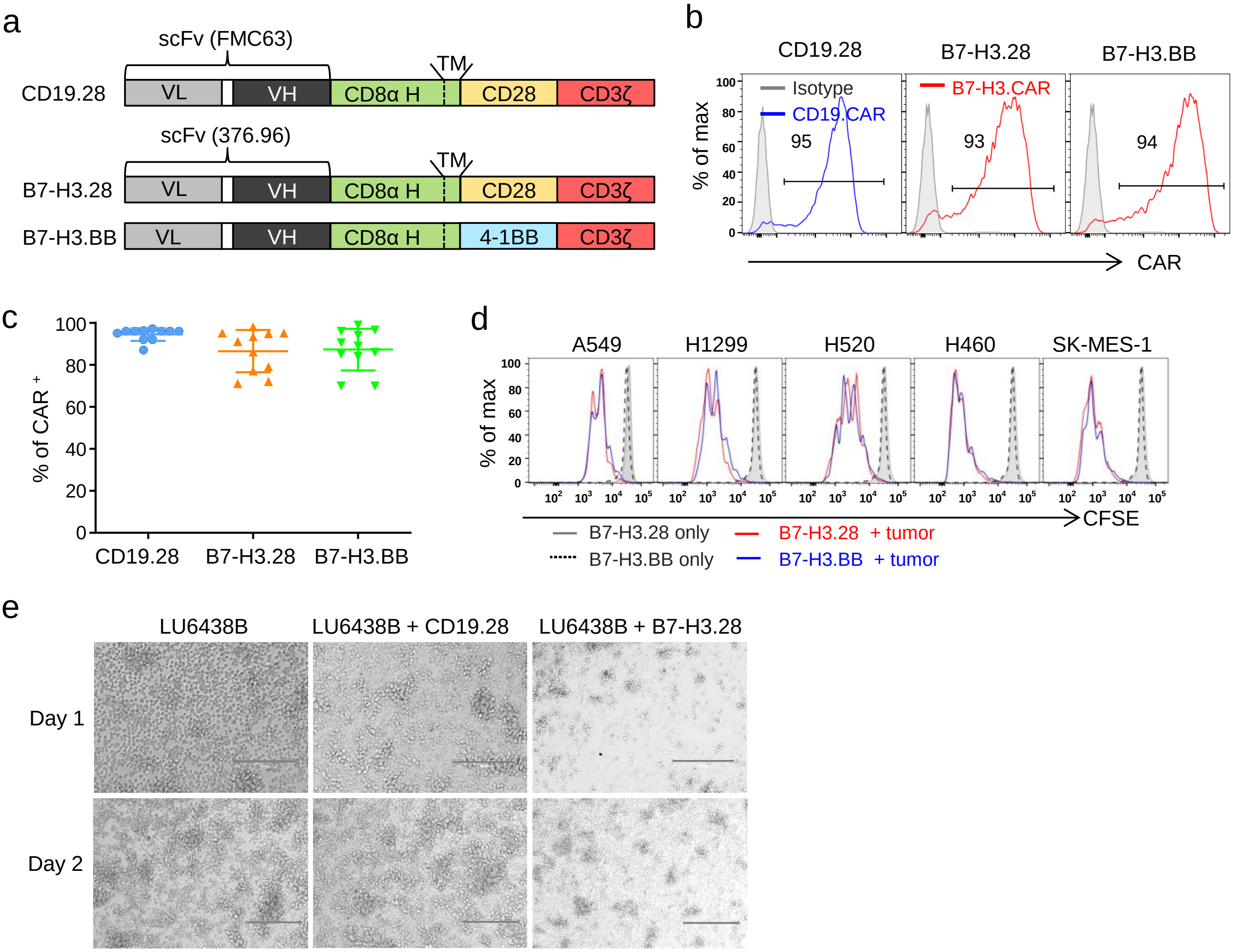

Supplementary Figure 1. CAR structure and functional characterization of CAR-Ts. (**a**) Schematic of the CD19.28, B7-H3.28 and B7-H3.BB CAR vectors. (**b**) Representative flow plots showing CAR expression of T cells transduced with CD19.28, B7-H3.28 or B7-H3.BB vectors. (**c**) Summary of CAR expression in CD19.28, B7-H3.28 and B7-H3.BB CAR-Ts (n = 11 independent experiments using CAR-T cells generated from 11 different donors). Data are presented as mean values +/- SD.(**d**) CFSE dilution of CFSE-labeled B7-H3.28 and B7-H3.BB CAR-T cells co-cultured with NSCLC cell lines for 5 days at 1 to 1 ratio analyzed by flow cytometry. CFSE-labeled CAR-T cells alone were used as control. (**e**) Representative microscopy images showing the lung cancer organoid LU6438B cocultured with CD19.28 or B7-H3.28 CAR-Ts at the CAR-T cells to organoid cells ratio of 1 to 2 after 24h and 48h of co-culture. This experiment was repeated two times independently with similar results. Scale bars, 300 µm. Source data for (**c**) are provided as a Source Data file.

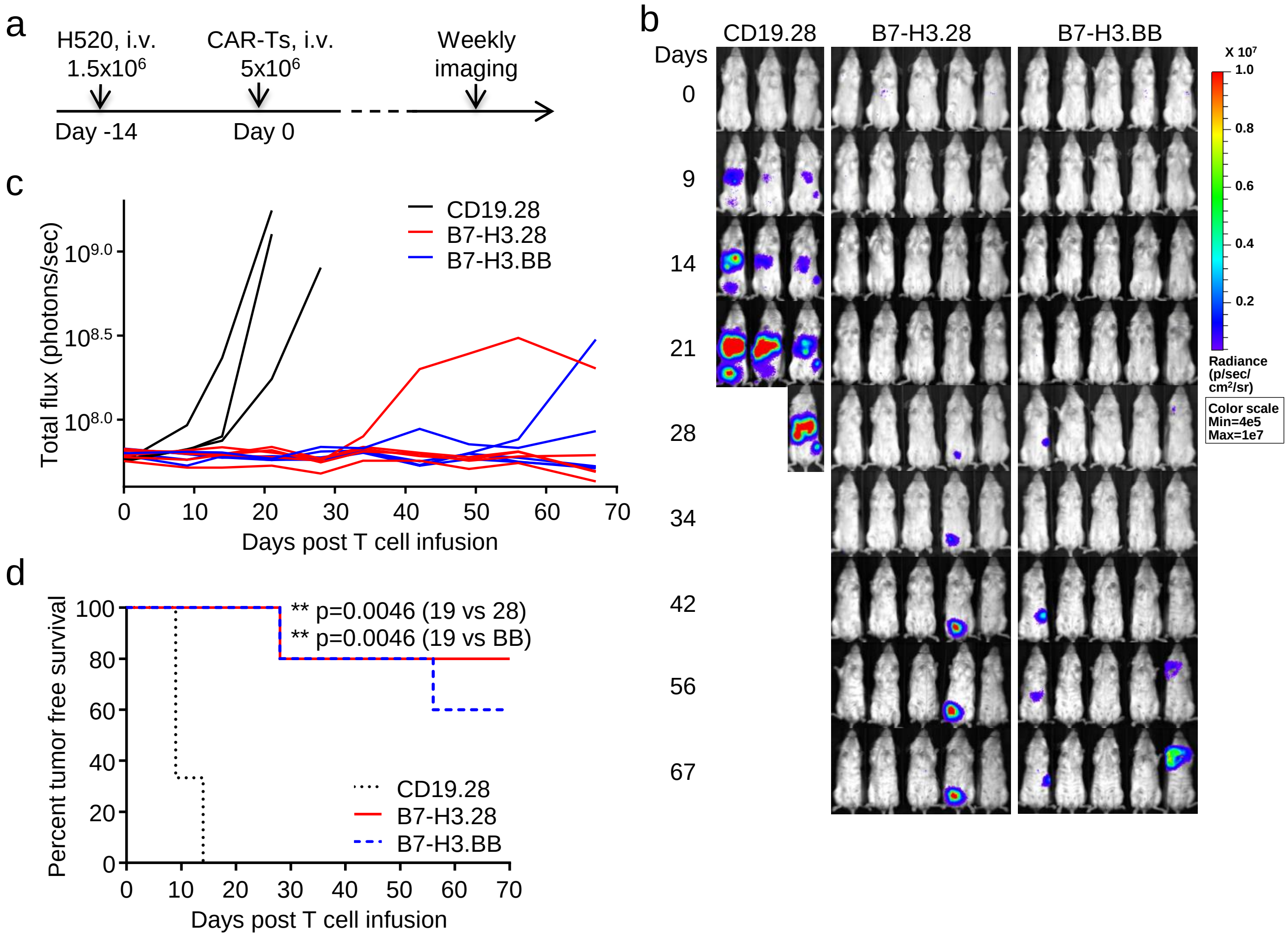

Supplementary Figure 2. B7-H3.CAR-Ts control B7-H3⁺ NSCLC in a metastatic model. (**a**) Schematic representation of the metastatic NSCLC model in which NSG mice are inoculated i.v. with FFluc-H520 cells and then treated with CAR-Ts by i.v. tail injection. Representative tumor images (BLI) (**b**) and kinetics (**c**) of tumor growth. (**d**) Kaplan-Meier tumor free survival curve of mice in (**a**), (n = 3 mice in CD19.28, n = 5 mice in B7-H3.28 and B7-H3.BB groups), **p = 0.0046 (B7-H3.28 vs. CD19.28), **p = 0.0046 (B7-H3.BB vs. CD19.28), chi-square test. Source data for (**c**, **d**) are provided as a Source Data file.

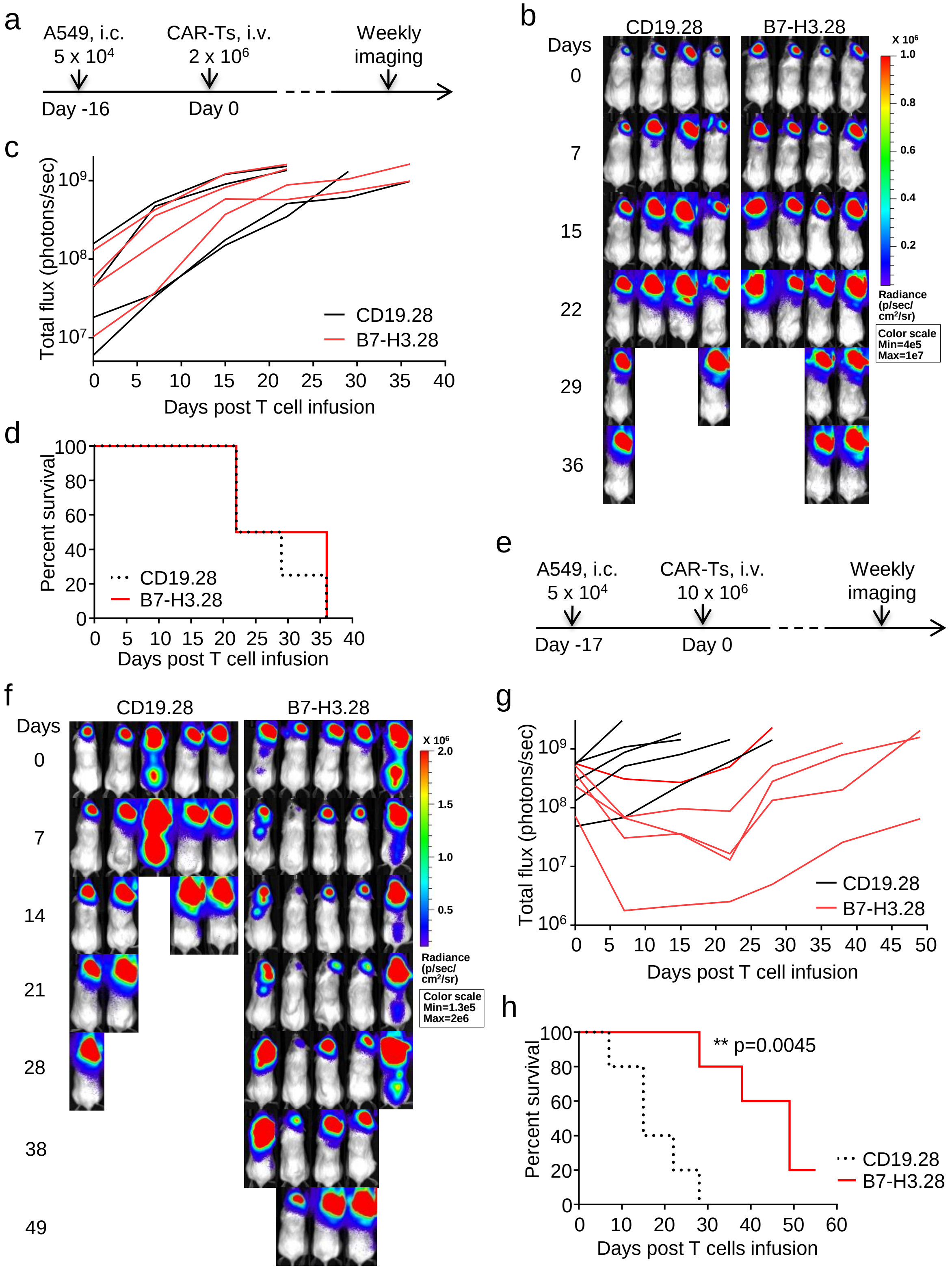

Supplementary Figure 3. B7-H3.CAR-Ts do not control A549 tumor growth in a brain xenograft model in NSG mice. (**a**) Schematic of the A549 brain tumor model in which NSG mice are implanted i.c. with FFluc-A549 cells (5×10^4 cells) and treated with low dose CD19.28 or B7-H3.28 CAR-T cells (2×10^6) 16 days after tumor implantation by i.v. injection. (**b** and **c**) Representative tumor BLI images (**b**) and BLI kinetics (**c**) of the A549 tumor growth in the model shown in (**a**). (**d**) Kaplan-Meier survival curves of mice in (**b**), n = 4 mice/group. (**e**) Schematic of the A549 brain tumor model in which NSG mice are implanted i.c. with FFluc-A549 cells (5×10^4 cells) and treated with high dose CD19.28 or B7-H3.28 CAR-T cells (10×10^6) 17 days after tumor implantation by i.v. injection. (**f** and **g**) Representative tumor BLI images (**f**) and BLI kinetics (**g**) of the A549 tumor growth in the model shown in (**e**). (**h**) Kaplan-Meier survival curves of mice in (**e**), **p = 0.0045, chi-square test, n = 5 mice/group. Days indicated in (**b, f**) represent the days post T cell infusion. Source data for (**c**, **d**, **g**, **h**) are provided as a Source Data file.

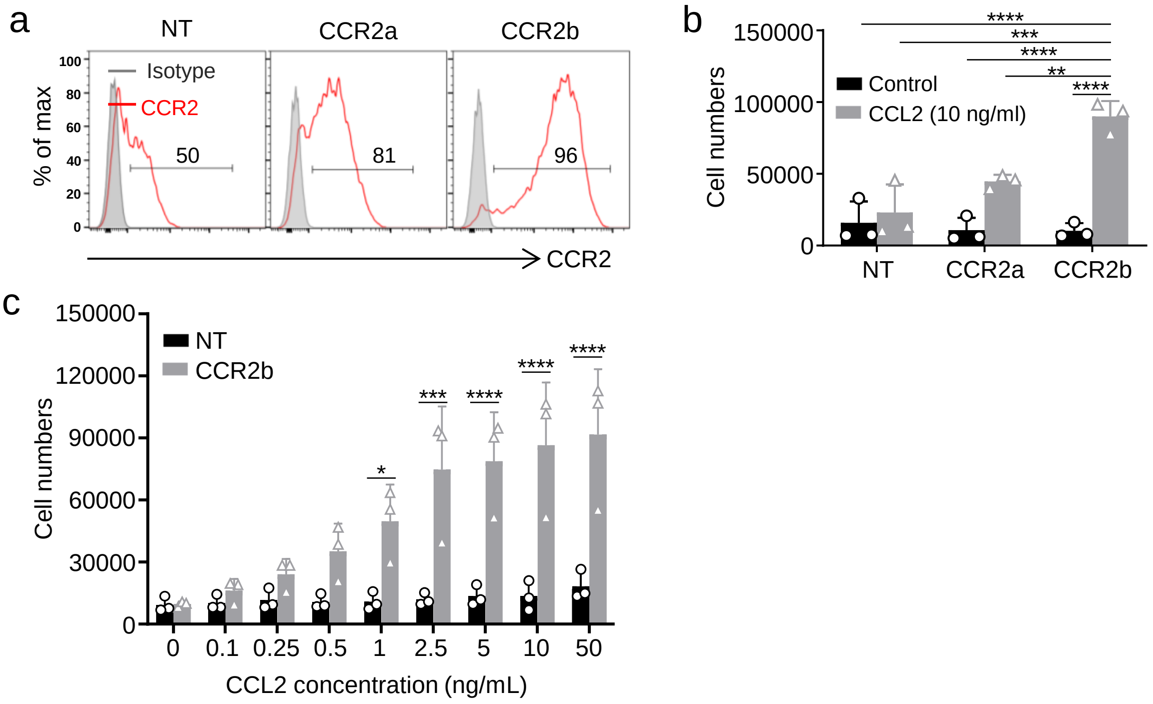

Supplementary Figure 4. CCR2b shows higher expression in T cells and promotes superior chemotaxis towards CCL2 compared with CCR2a. (**a**) Representative flow plots showing CCR2 expression in T cells transduced with vectors encoding human CCR2a or CCR2b. (**b**) Counting of T cells migrating in response to CCL2 in transwell assays when T cells express either CCR2a or CCR2b (n = 3 independent experiments using CAR-T cells generated from 3 different donors), **p = 0.0081, ***p = 0.0002, ****p < 0.0001, two-way ANOVA with Sidak's multiple comparisons test. Data are presented as mean values + SD. (**c**) Counting of T cells expressing CCR2b and migrating in transwell assays in response to different concentrations of CCL2 (n = 3 independent experiments using CAR-T cells generated from 3 different donors), error bars denote SD. *p = 0.0326, ***p = 0.0001, ****p < 0.0001, two-way ANOVA with Sidak's multiple comparisons test. Data are presented as mean values + SD. Source data for (**b**, **c**) are provided as a Source Data file.

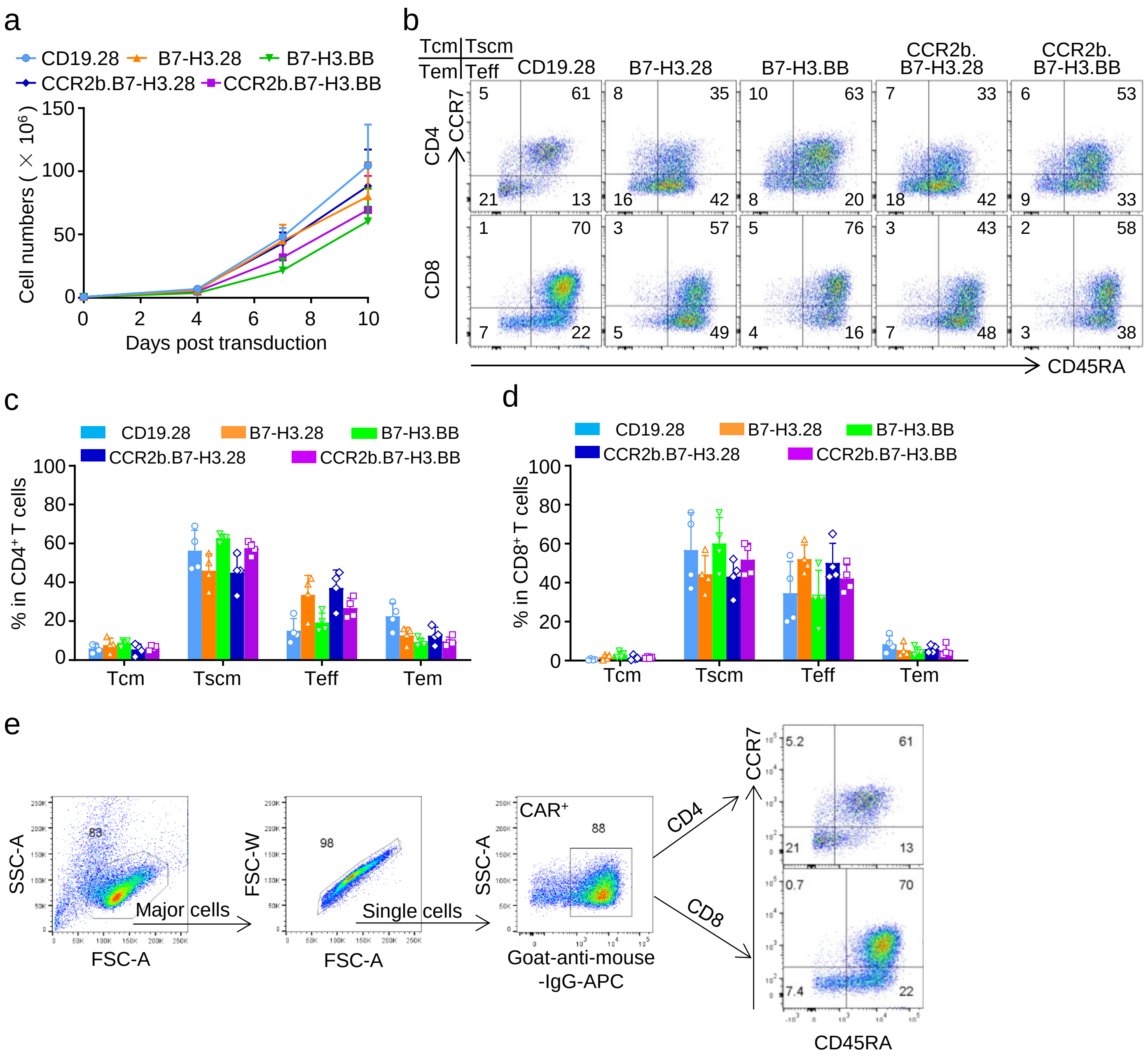

Supplementary Figure 5. CCR2b co-expression in B7-H3.CAR-Ts does not impair cell expansion and phenotypic profile. (**a**) T cell expansion in vitro of CD19.28, B7-H3.28, B7-H3.BB, CCR2b.B7-H3.28 and CCR2b.B7-H3.BB CAR-T cells. Days indicated represent the days post T cell transduction. Data are presented as mean values + SD (n = 4 independent experiments using CAR-T cells generated from 4 different donors). (**b**) Flow-cytometry plots showing the memory phenotype profile (Tcm, Tscm, Tem and Teff) in CD4$^+$ or CD8$^+$ T cells in NT, and CAR-T cells. T cells analyzed on day 12 post T cell transduction. (**c** and **d**) Summary of Tcm, Tscm, Tem and Teff cells in CD4$^+$ or CD8$^+$ cells. Data are presented as mean values + SD (n = 4 independent experiments using CAR-T cells generated from 4 different donors). (**e**) Representative flow gating strategy for the memory phenotype of the CAR-T cells showed in (**b-d**). Source data for (**a**, **c**, **d**) are provided as a Source Data file.

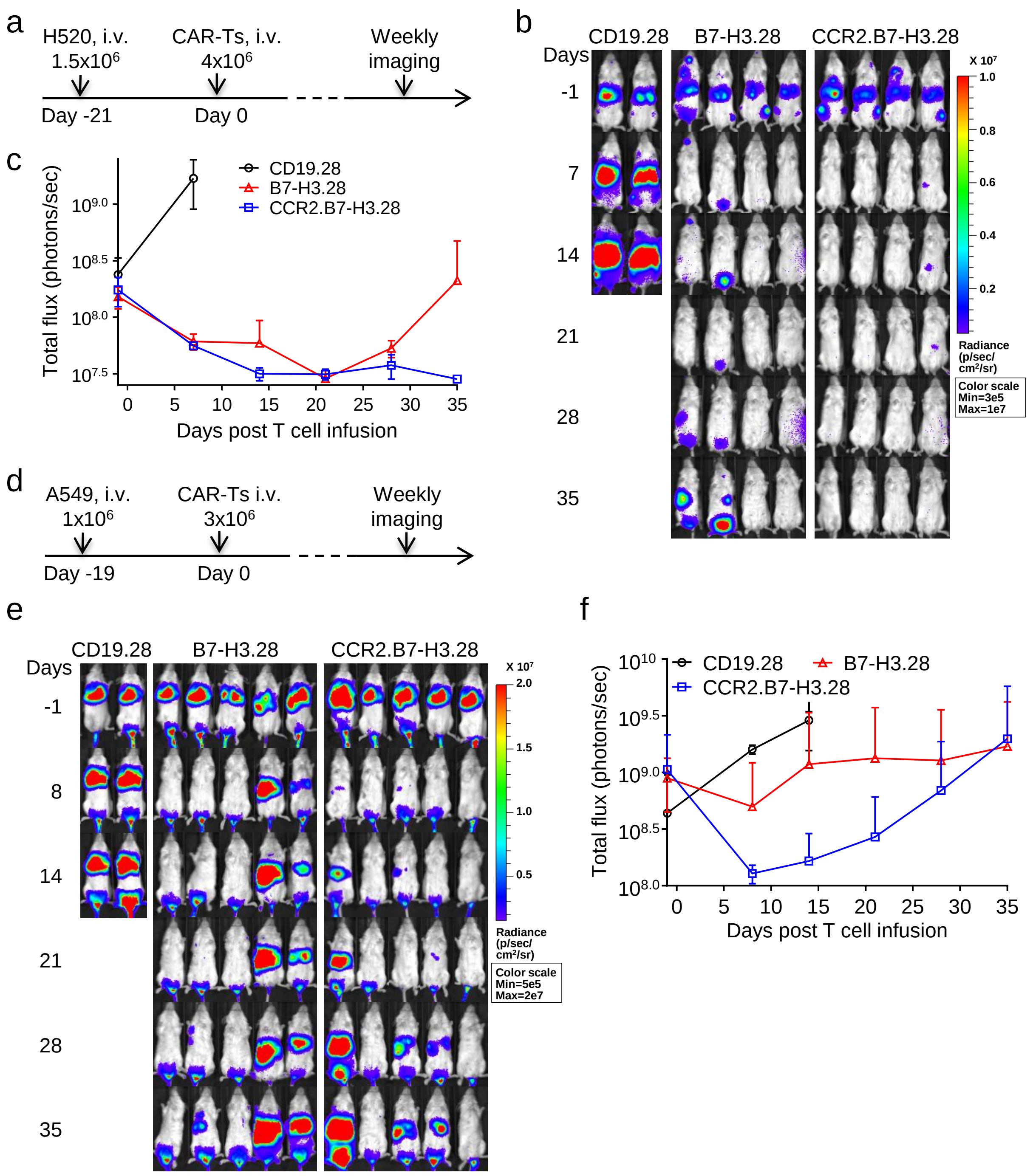

Supplementary Figure 6. CCR2b co-expression in B7-H3.CAR-Ts modestly enhances their antitumor activity in NSCLC metastatic models. (**a-c**) Schematic representation of the metastatic NSCLC model in which NSG mice are inoculated i.v. with FFluc-H520 cells and then treated with CD19.28, B7-H3.28, or CCR2b.B7-H3.28 CAR-T cells by i.v. injection. (**a**) Schematic representation of the H520 metastatic model. Representative images of tumor bioluminescence (BLI) (**b**) and kinetics (**c**) of tumor growth as assessed by BLI measurements, data are presented as mean values + SD in (**c**) (n = 2 mice in CD19.28, n = 4 mice in B7-H3.28 and CCR2b.B7-H3.28). (**d-f**) Metastatic model of A549 cell line in NSG mice. (**d**) Schematic representation of the metastatic NSCLC model in which NSG mice are inoculated i.v. with FFluc-A549 cells and then treated with CD19.28, B7-H3.28, or CCR2b.B7-H3.28 CAR-T cells. Representative images of tumor BLI (**e**) and kinetics (**f**) of tumor growth as assessed by BLI measurements, data are presented as mean values + SD in (**f**) (n = 2 mice in CD19.28, n = 5 mice in B7-H3.28 and CCR2b.B7-H3.28). Days indicated in (**b**) and (**e**) represent the days post T cell infusion. Source data for (**c**, **f**) are provided as a Source Data file.

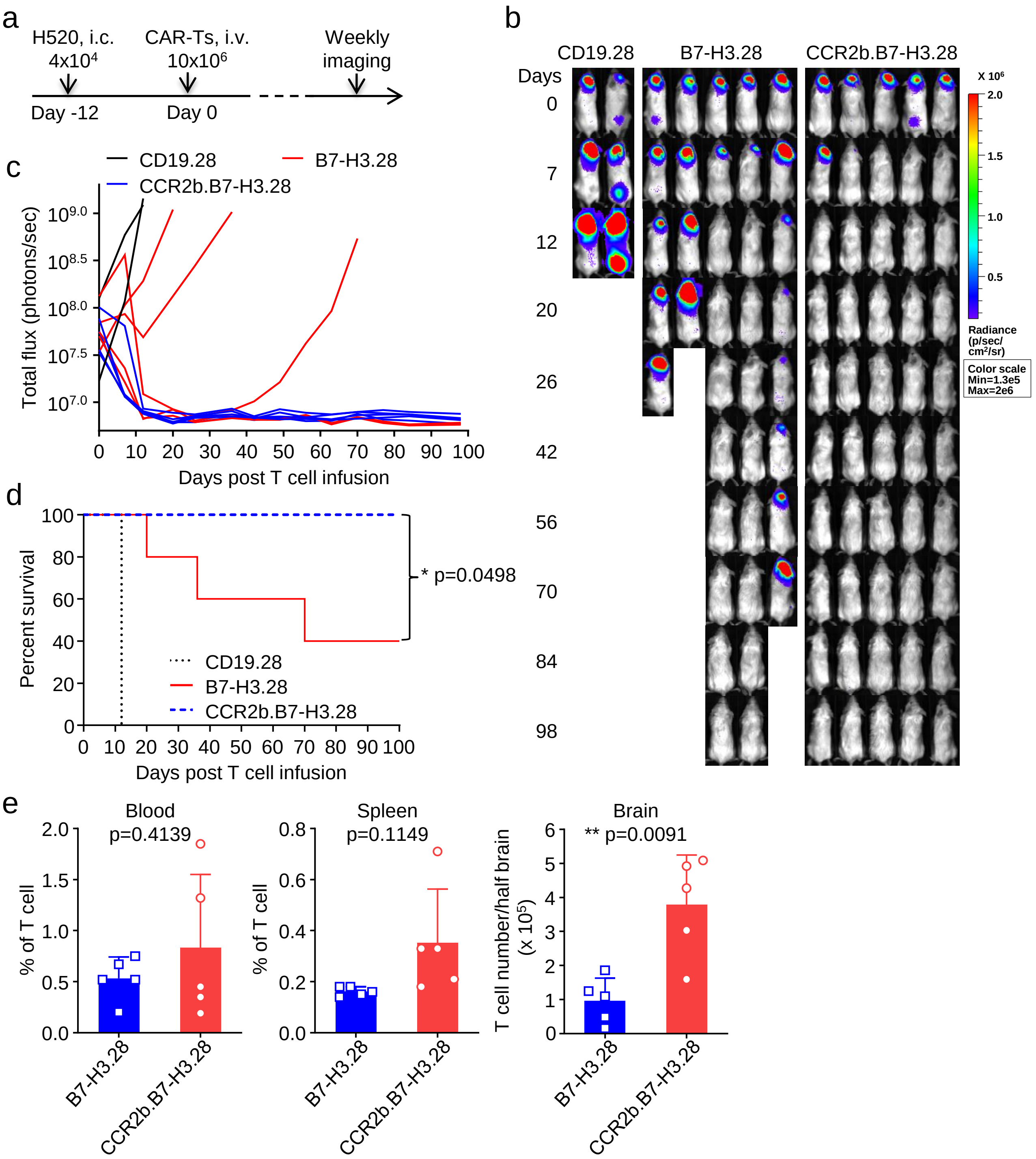

Supplementary Figure 7. CCR2b co-expressing B7-H3.CAR-Ts show superior antitumor activity in the H520 brain tumor model. (**a**) Schematic of the H520 brain tumor model in which NSG are implanted i.c. with FFluc-H520 cells (4×10^4 cells) and treated with CD19.28, B7-H3.28 or CCR2b.B7-H3.28 CAR-T cells (10×10^6) injected i.v. 12 days later. (**b** and **c**) Representative tumor BLI images (**b**) and BLI kinetics (**c**) of the FFluc-H520 tumor growth in the model shown in (**a**). Days indicated in (**b**) represent the days post T cell infusion. (**d**) Kaplan-Meier survival curves of mice in (**b**), *p = 0.0498, chi-square test. N = 2 mice in CD19.28, n = 5 mice in B7-H3.28 and CCR2b.B7-H3.28 in (**c** and **d**). (**e**) In the separate experiment, the mice were euthanized 8 days after CAR-T cells infusion, and human $CD45^+CD3^+$ T cells were quantified in blood, spleen and tumor by flow cytometry. Bar graph summaries are shown (n = 5 mice/group). Data are presented as mean values + SD; **p = 0.0091, unpaired t test with Welch's correction and two-tailed p value calculation. Source data for (**c**, **d**, **e**) are provided as a Source Data file.

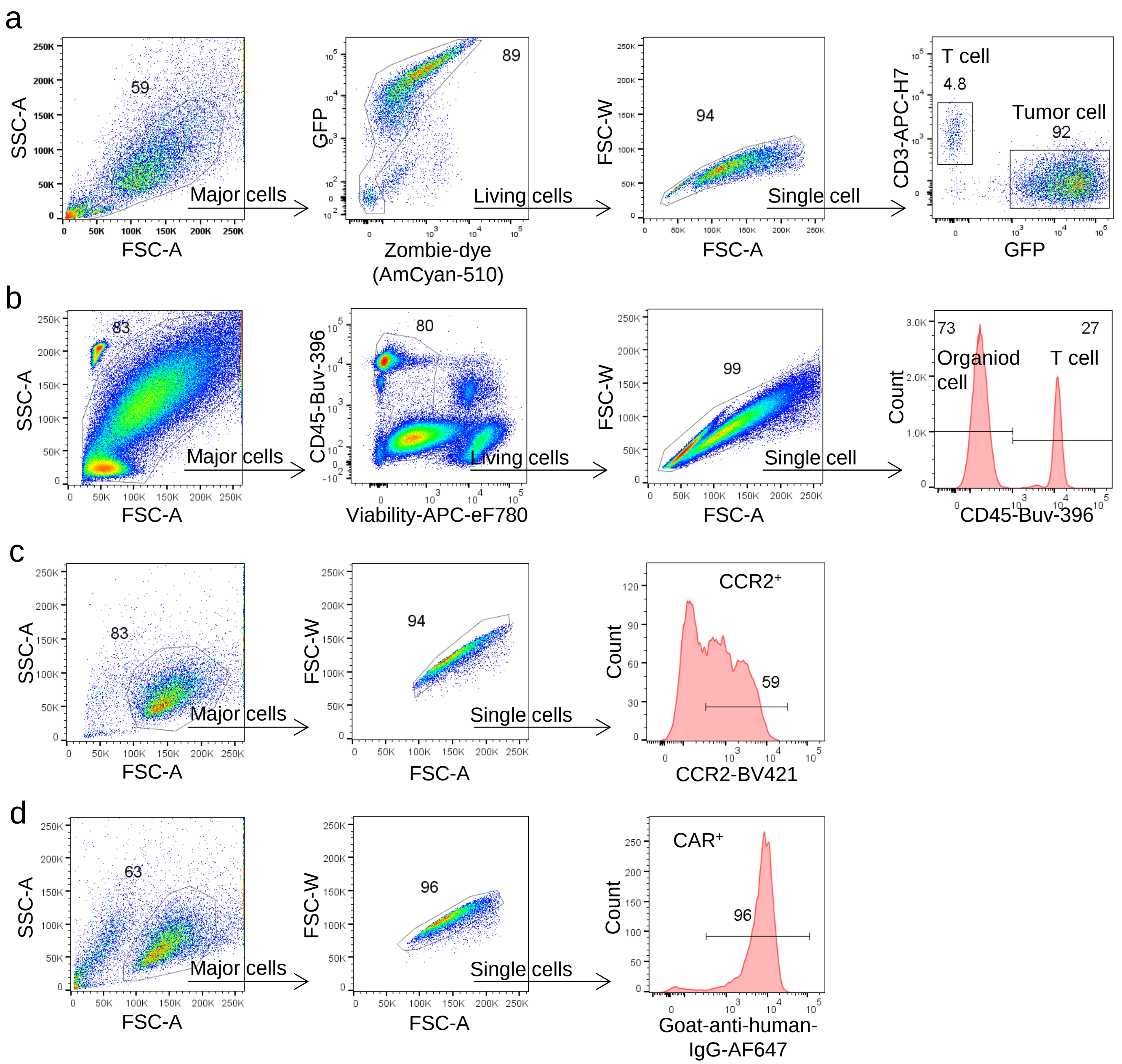

Supplementary Figure 8. Gating strategies. (**a**) Representative gating strategy for flow cytometry analysis of the co-culture experiments with NSCLC tumor cell lines for Fig. 1d-e and Fig 4d-e. (**b**) Representative gating strategy for flow cytometry analysis of the co-culture experiments with organoid cells for Fig. 1i-j. (**c**) Representative gating strategy for flow cytometry analysis of the CCR2 expression in Fig. 3e-f and Supplementary Fig. 4a. (**d**) Representative gating strategy for flow cytometry analysis of the CAR expression in Fig. 4a-c and Supplementary Fig. 1b-c.

Supplementary Table 1. Summary of chemokine genes expression in lung cancer

Gene	Brain (MD)	Lung (MD)	LUAD (TCGA)	LUSC (TCGA)
CXCL2	9.877215	10.77373	8.51883	7.085441
CXCL1	9.234862	9.520319	7.979941	9.084662
CXCL16	8.893182	8.958561	11.36734	10.49259
CCL2	8.547023	9.242671	9.421721	9.112555
CCL18	8.484626	10.30898	10.26809	9.912602
CCL20	8.007272	8.134597	7.956865	7.646065
CCL5	7.90908	9.474124	9.61017	9.482056
CXCL12	7.868033	8.422825	9.194566	8.793509
CCL4	7.833945	7.914497	7.310597	7.19234
CXCL14	7.77247	8.338863	9.225043	10.30839
CCL3L1	7.747769	7.312868	4.438269	4.419468
CCL3	7.714433	7.285501	7.204345	7.043956
CXCL9	7.683731	10.49648	9.946797	9.648854
CXCL3	7.593487	7.701252	5.644422	5.277359
CXCL10	6.422961	7.148977	8.628386	8.889078
CXCL13	6.394719	8.37715	8.336519	8.616292
CXCL6	6.241834	5.684184	4.716799	6.771357
CCL13	6.07379	7.448635	7.203175	5.752633
CXCL5	5.976363	5.588311	6.583589	5.725901
CXCL11	5.793083	6.67535	6.27315	6.164005
CCL14	5.758235	7.356038	6.861608	5.487592
CCL19	5.753628	9.717836	7.893972	7.357034
CCL8	5.319558	5.131602	5.611836	5.241191
CCL22	5.254654	5.76306	7.533177	6.750719
CCL17	5.17511	5.328044	4.640333	3.373005
CCL26	4.770659	4.647026	2.411255	4.356511
CCL28	4.66718	3.648037	5.778392	5.401857
CCL7	4.256297	3.846952	3.314135	2.875969
CCL27	4.013291	3.230837	-0.74517	-0.00848
CCL15	3.639623	3.06721	2.768091	1.289302
CCL21	3.557192	8.834228	9.173221	8.777382
CCL16	3.399015	3.287316	0.281874	-0.12553
CCL23	3.353734	3.951275	3.465539	2.09642
CCL11	3.287908	5.747951	4.478769	5.385724
CCL25	3.20205	2.926472	1.094847	0.869083
CCL1	3.096825	3.31948	0.321498	-0.09343
CCL24	2.884338	2.904312	1.649996	1.58738
CXCL17	NA	NA	11.6394	9.336873
CCL4L2	NA	NA	6.308443	6.279797
CCL15	NA	NA	3.26691	2.185574
CCL3L3	NA	NA	1.770742	1.47255

Mean gene expression level (log2) of CCL and CXCL chemokine family members (41 genes) in 37 primary lung cancer (Lung-MD) paired with brain metastatic lesions (Brain-MD) from MD Anderson Cancer Center data set, and in lung cancer adenocarcinoma (LUAD) (n = 517) and lung cancer squamous cell carcinoma (LUSC) (n = 501) cohorts from TCGA data set. The data was sorted by the expression level in the brain metastatic lesions from MD Anderson Cancer Center cohorts.
