# Supplementary material for: Targeting brain lesions of non-small cell lung cancer by enhancing CCL2-mediated CAR-T cell migration
Source: Nat Commun. 2022 Apr 20;13:2154. doi: 10.1038/s41467-022-29647-0 (PMC9021299; doi:10.1038/s41467-022-29647-0)
Supplement: Supplementary file 2 — Reporting Summary [file 41467_2022_29647_MOESM2_ESM.pdf]

nature portfolio

Corresponding author(s): Gianpietro Dotti and Hongwei Du

Last updated by author(s): Mar 15, 2022

Reporting Summary

Nature Portfolio wishes to improve the reproducibility of the work that we publish. This form provides structure for consistency and transparency in reporting. For further information on Nature Portfolio policies, see our Editorial Policies and the Editorial Policy Checklist.

Statistics

For all statistical analyses, confirm that the following items are present in the figure legend, table legend, main text, or Methods section.

n/a	Confirmed	
☐	☒	The exact sample size (*n*) for each experimental group/condition, given as a discrete number and unit of measurement
☐	☒	A statement on whether measurements were taken from distinct samples or whether the same sample was measured repeatedly
☐	☒	The statistical test(s) used AND whether they are one- or two-sided<br>*Only common tests should be described solely by name; describe more complex techniques in the Methods section.*
☐	☒	A description of all covariates tested
☐	☒	A description of any assumptions or corrections, such as tests of normality and adjustment for multiple comparisons
☐	☒	A full description of the statistical parameters including central tendency (e.g. means) or other basic estimates (e.g. regression coefficient) AND variation (e.g. standard deviation) or associated estimates of uncertainty (e.g. confidence intervals)
☐	☒	For null hypothesis testing, the test statistic (e.g. *F*, *t*, *r*) with confidence intervals, effect sizes, degrees of freedom and *P* value noted<br>*Give P values as exact values whenever suitable.*
☒	☐	For Bayesian analysis, information on the choice of priors and Markov chain Monte Carlo settings
☒	☐	For hierarchical and complex designs, identification of the appropriate level for tests and full reporting of outcomes
☒	☐	Estimates of effect sizes (e.g. Cohen's *d*, Pearson's *r*), indicating how they were calculated

Our web collection on statistics for biologists contains articles on many of the points above.

Software and code

Policy information about availability of computer code

Data collection	Flow cytometry data were collected on BD LSR Fortessa and BD Canto II using BD FACS DIVA software v8.0.1 (all BD). ELISA data was collected with Synergy 2 Multi-Detection Microplate Reader with Gen5 v2.0.7 software (both BioTek). Tumor growth in mice was monitored by bioluminescence imaging using the IVIS lumina II in vivo imaging system with Living Image software v4.5.2 (both PerkinElmer) or AMI Optical in vivo imaging system with Aura software v2.3.1 (Spectral instruments imaging).
Data analysis	Flow cytometry data was analyzed using Flowjo v10. GraphPad Prism v8 is a graphing software that was used for figure generation and statistical analyses. Expression data of 41 CCL and CXCL family members genes were log2 transformed and subjected to density estimation and plotted using RStudio v1.2.5.

For manuscripts utilizing custom algorithms or software that are central to the research but not yet described in published literature, software must be made available to editors and reviewers. We strongly encourage code deposition in a community repository (e.g. GitHub). See the Nature Portfolio guidelines for submitting code & software for further information.

Data

Policy information about availability of data

All manuscripts must include a data availability statement. This statement should provide the following information, where applicable:

- Accession codes, unique identifiers, or web links for publicly available datasets
- A description of any restrictions on data availability
- For clinical datasets or third party data, please ensure that the statement adheres to our policy

Source data are provided with this paper. H&E staining and SNP analysis data of the lung cancer organoid models can be accessed for free through Crownbio organoid database (https://organoid.crownbio.com).

Field-specific reporting

Please select the one below that is the best fit for your research. If you are not sure, read the appropriate sections before making your selection.

- [x] Life sciences
- [ ] Behavioural & social sciences
- [ ] Ecological, evolutionary & environmental sciences

For a reference copy of the document with all sections, see nature.com/documents/nr-reporting-summary-flat.pdf

Life sciences study design

All studies must disclose on these points even when the disclosure is negative.

Sample size	Sample sizes for experiments were estimated based on previous experience and literature. All in vitro experiments were repeated at least three separate times (three different T cell donors). For in vivo experiments using NSG mice, all experiments were repeated using at least two different T cell donors with at 4-5 mice in each group (8-10 mice/group in total).
Data exclusions	No data was excluded.
Replication	For in vitro experiments, such as CAR expression efficiency and co-culture experiments, the experiments were repeated by using CAR-T cells generated from at least 3 different donors (3-10), each donor of CAR-T cell indicate one repeat. For in vivo mouse experiment, all the experiments were repeated using at least two different T cell donors with at 4-5 mice in each group (8-10 mice/group in total), each mice represents one repeat. The replication of experiments showed similar results or trend.
Randomization	Samples and organisms were randomly allocated to groups for in vitro and in vivo experiments.
Blinding	The experiments were not performed in blind. Blinding is not feasible in the experiments described here due to the treatment and follow up schedule.

Reporting for specific materials, systems and methods

We require information from authors about some types of materials, experimental systems and methods used in many studies. Here, indicate whether each material, system or method listed is relevant to your study. If you are not sure if a list item applies to your research, read the appropriate section before selecting a response.

Materials & experimental systems

n/a	Involved in the study
[ ]	[x] Antibodies
[ ]	[x] Eukaryotic cell lines
[x]	[ ] Palaeontology and archaeology
[ ]	[x] Animals and other organisms
[ ]	[x] Human research participants
[x]	[ ] Clinical data
[x]	[ ] Dual use research of concern

Methods

n/a	Involved in the study
[x]	[ ] ChIP-seq
[ ]	[x] Flow cytometry
[x]	[ ] MRI-based neuroimaging

Antibodies

Antibodies used	The following antibodies used for the flow cytometry analysis were purchased from BD Biosciences: APC-H7-conjugated anti-CD3 (Clone SK7; Cat#: 560176; lot: 1021392; dilution: 1:100), BV421-conjugated anti-CD4 (Clone RPA-T4; Cat#: 562424; lot: 0084457; dilution: 1:100), APC-conjugated anti-CD8 (clone SK1; Cat#: 340584; lot: 340584; dilution: 1:100), CD45-BV510 (Clone HI30; Cat#: 563204; lot: 9344066; dilution: 1:30), CD45RA-PE (Clone HIO0; Cat#: 555489; lot:9246697; dilution: 1:30), CCR7-FITC (clone 150503; Cat#: 561271; lot: 9179069; dilution: 1:50), BV421-conjugated anti-B7-H3 (clone 7-517; Cat#: 565829; lot: 1111225; dilution: 1:100). BV421-conjugated-CCR2 (clone K036C2; Cat#: 357210; lot: B325976; dilution: 1:100) was purchased from Biolegend.<br>Anti-CD19-CAR idiotype (Clone 233-4A, dilution: 1:100) and Anti-B7-H3 mAb (clone 376.96, dilution: 1:100) were purified from the corresponding hybridoma cell culture supernatant by UNC protein purification core.<br>APC-conjugated-goat-anti-mouse Ig (BD Biosciences, polyclonal, Cat#: 550826; dilution 1:100), AF647-conjugated-goat-anti-human IgG (H+L) Ab (Jackson ImmunoResearch Laboratories Inc., polyclonal, Cat# 109-606-088, dilution 1:100).<br>EnVision+ System-HRP-Labelled-Polymer Anti-mouse IgG for IHC staining was purchased from Agilent Dako (code: K4001, used without further dilution).
Validation	All antibodies used were validated by the suppliers for their particular application, and also validated in the lab by staining of the cells with or without the specific antigen expression. Validation statements and relevant references for the used antibodies can be found on the manufacturer's website.

Eukaryotic cell lines

Policy information about cell lines

Cell line source(s)	Human non-small cell lung cancer (NSCLC) cell lines A549 (Cat# CCL-185), H1299 (Cat# CRL-5803) and SK-MES-1 (Cat# HTB-58) were originally purchased from American Type Culture Collection (ATCC); H460 (Cat# HTB-177) and H520 (Cat# HTB-182) were obtained from the Tissue Culture Facility of University of North Carolina at Chapel Hill, originally purchased from American Type Culture Collection (ATCC).
Authentication	All cell lines were routinely examined and confirmed by the morphology and growing behaviors under microscopy (adherent, growth rate, etc.), which are consistent with the phenotype showed in ATCC. Since the cells were originally from ATCC, we have not done extra authentication by using the technologies of STR profiling, Karyotyping, DNA barcoding, PCR assays.
Mycoplasma contamination	Mycoplasma test is performed biweekly in the lab, all cell lines were mycoplasma free.
Commonly misidentified lines (See ICLAC register)	None of cell line used are commonly misidentified lines based on ICLAC register V9.

Animals and other organisms

Policy information about studies involving animals; ARRIVE guidelines recommended for reporting animal research

Laboratory animals	Female 4 - 6 weeks old NSG mice were purchased from the Animal Core Facility at University of North Carolina at Chapel Hill (UNCCH in-house breeding), and housed in the Animal Core Facility at UNC until the age ready for experiment (8-12 weeks old).
Wild animals	No wild type animals were used in the study.
Field-collected samples	No Field-collected samples were used in the study.
Ethics oversight	All mouse experiments were performed in accordance with UNC Animal Husbandry and Institutional Animal Care and Use Committee (IACUC) guidelines and were approved by UNC IACUC. All animal experiments were conducted in compliance with all relevant ethical regulations.

Note that full information on the approval of the study protocol must also be provided in the manuscript.

Human research participants

Policy information about studies involving human research participants

Population characteristics	Human healthy donors PBMCs was purchased from the Gulf Coast Regional Blood Center (GCRBC, Houston, TX), the donors' information was deidentified by GCRBC. Thus, the characteristics are unknown for us.
Recruitment	The human PBMCs were collected, processed and distributed by the Gulf Coast Regional Blood Center (Houston, TX). Therefore, our lab or institute does not involved in any process of the donor recruitment.
Ethics oversight	Human PBMCs was purchased from the Gulf Coast Regional Blood Center (GCRBC, Houston, TX), the donors' information was deidentified by GCRBC. Thus, the use of the PBMCs does not require Institutional Review Board (IRB) approval.

Note that full information on the approval of the study protocol must also be provided in the manuscript.

Flow Cytometry

Plots

Confirm that:

- [x] The axis labels state the marker and fluorochrome used (e.g. CD4-FITC).
- [x] The axis scales are clearly visible. Include numbers along axes only for bottom left plot of group (a 'group' is an analysis of identical markers).
- [x] All plots are contour plots with outliers or pseudocolor plots.
- [x] A numerical value for number of cells or percentage (with statistics) is provided.

Methodology

Sample preparation	For surface staining, cells were incubated with antibodies at room temperature for 10 min or at refrigerator for 20 min. In most assays, cells were stained with Zombie Aqua Live/Dead Discrimination dye (Biolegend) to gate out dead cells for analysis.
Instrument	Flow cytometry data were collected on BD LSR Fortessa and BD Canto II using BD FACS DIVA software v8.0.1.
Software	BD FACS DIVA software v8.0.1 and data was analyzed by using Flowjo v10.

Cell population abundance

No sorting was performed in this study.

Gating strategy

For all experiments, FSC-A/SSC-A gates of the starting cell population were used to discriminate between viable cells and cell debris. Singlet and doublet cells were discriminated using FSC-W/ FSC-A gating. For the co-culture expreiments, Live cells were further selected by gating out the dead cells by Zombie Aqua Dye (Biolegend) staining. For dertimining the CAR-T cell persistence in mouse experiments, the human CAR-T cell population was firstly selected by gating on the human CD45 marker positive population, and then used for downstream analysis. Generally, the boundary used to distinguish the negative and positive staining cell populations is 10e2. Further gating strategy information and figures can be provided upon request.

☒ Tick this box to confirm that a figure exemplifying the gating strategy is provided in the Supplementary Information.
